# The Role of the Evacuation Hospital in the Care of the Wounded

**Published:** 1919

**Authors:** 


***The Role of the Evacuation Hospital in the Care of the Wounded.*** By Daniel Fiske Jones, M. D., *Annals of Surgery*, August, 1918.

The considerable importance of early treatment of wounds, and the necessity of treating the wounded within ambulance drive from the front, does not seem yet to be sufficiently realized by such medical officers as have not come into contact with the gas bacillus and streptococcus.

The following was the author's experience: The general Hospital No. 22, B.E.F., a two-thousand-bed hospital, and hospital after hospital of the same size, were filled with practically nothing but cases of wounds infected with the gas bacillus or streptococcus, and the average time of arrival at the hospital was 3 days after being wounded. Men were sent to England after from 3 days to 6 weeks, with many more wounds than they came with, all discharging pus freely, and with no suggestion of bony union in compound fractures. Whereas in another ward, filled with patients operated upon within 12 hours of receipt of the wound, and then treated by the Carrel-Dakin method, about 80 per cent. of the wounds were closed successfully within 10 days. It is concluded, therefore, that all wounds should be cared for as early as possible; that is, at the casualty clearing station, which corresponds to the evacuation hospital.

Such units were practically non-existent before 1916; they have been developed considerably since then. They are now a nucleus about which a great mobile force of operating teams, nurses and orderlies can be gathered in a comparatively few hours, when the battle has begun in some sector.

The evacuation hospital must be a combination of field and base hospitals. In location and in the early treatment of the wounded it may be considered as a field hospital; in size, permanency, equipment, and the work it does, it is really a base hospital.

The author insists on the necessity of having most experienced surgeons in the evacuation hospital. He considers that the value of the casualty clearing stations in 1916 was practically nullified, mostly because of inexperienced surgeons.

To make the evacuation hospital most effective, the patient should arrive there within 12 hours, and the staff must be made up of surgeons of ability and experience. An effort has been made to keep the experienced men in the base hospitals down to 40 per cent. of the staff, but the author considers it of still greater importance to keep the experienced men, who are to work at the evacuation hospital, up to 100 per cent.

The comparison of the course of a patient with an infected compound thigh fracture with splinting of several inches of the femur, and the course of a similar case properly cleaned with the wound immediately closed, or closed in ten days, this being multiplied by many thousands, gives an idea of the value of evacuation hospitals when properly equipped, and with permanent staffs of experienced surgeons, aided by mobile units and operating teams from various base and field hospitals and inactive evacuation hospitals.

